# Oral hygiene aspects in a study of children and young adults with the congenital and childhood forms of myotonic dystrophy type 1

**DOI:** 10.1002/cre2.36

**Published:** 2016-08-04

**Authors:** Åsa Mårtensson, Anne‐Berit Ekström, Monica Engvall, Lotta Sjögreen

**Affiliations:** ^1^ Mun‐H‐Center Orofacial Resource Centre for Rare Diseases Public Dental Service Gothenburg Sweden; ^2^ Department of Pediatrics, Institute of Clinical Sciences, The Queen Silvia Children's Hospital Sahlgrenska Academy at the University of Gothenburg Sweden; ^3^ Department of Pedodontics, Institute of Odontology The Sahlgrenska Academy at Gothenburg University Gothenburg Sweden

**Keywords:** Dental, disability, myotonic dystrophy, oral care, oral health, oral hygiene

## Abstract

The primary aim was to study the interaction between oral hygiene, oral care, saliva production, and oral motor function in individuals with myotonic dystrophy type 1 (DM1). A secondary aim was to study how oral hygiene, oral care, and saliva flow rate are affected by gender, age, and subgroup of DM1 in this study population. The study comprised 52 individuals, seven to 29 years of age, divided into two subgroups of DM1, the congenital (*N* = 24) and childhood‐onset forms (*N* = 28). A combined dental and oral motor examination was performed and the participants or caregivers answered a questionnaire with questions about general health and disabilities, medication, dental care, and oral health. Sixteen individuals with a plaque‐, gingivitis‐, or calculus‐index score of 5–6 were considered to have poor oral hygiene. There were no significant differences between subgroups (age, gender, or form of DM1) in terms of the occurrence of calculus, gingivitis, plaque, or saliva flow rate. The mean value of the unstimulated whole saliva flow rate was 0.7(±0.44) mL/min. An open mouth at rest and oral motor dysfunction were frequent findings. The majority of Swedish children and young adults with the congenital or childhood form of DM1 have fair or poor oral hygiene, with a high occurrence of plaque and gingivitis. As a group, individuals with DM1 and poor oral hygiene have a higher frequency of caries and they report less satisfaction with their oral care at home and the quality of dental care received compared with those with good oral hygiene.

## Background

Myotonic dystrophy type 1 (DM1) is a multisystemic disorder with an autosomal dominant inheritance caused by an expansion of a CTG trinucleotide repeat in the 3′ untranslated region of the DMPK‐gene on chromosome 19q13.3. The disorder results in the slow progression of muscular weakness. The age at onset, as well as presenting symptoms, is variable and the disorder is categorized into different subforms based on the age at which the individual experiences the first symptoms: congenital, childhood, adult, and late onset. The earlier the onset of the disease, the more serious the symptoms (Harper 2001). Intellectual disability and neuropsychiatric disorders are common in congenital and childhood subforms of DM1 (Ekström et al. 2008).

Engvall et al. (2007) have previously shown that the oral health of individuals with the congenital and childhood forms of DM1 was affected to a significantly greater degree compared with healthy controls. Intensified prophylactic dental care was often indicated and, because of neuropsychiatric and neurodevelopmental diagnoses, some individuals required general anesthesia for dental treatment. When the same patients grew older, the risk of having plaque, gingivitis, caries, and temporomandibular disorders increased (Engvall et al. 2009). The reasons behind the increased risk of periodontal disease in DM1 have not yet been fully elucidated. Engvall et al. (1991) suggested that the higher frequency of plaque and gingivitis in adults with the classical form of DM1 could be because of motor dysfunction. A positive correlation between finger force and oral hygiene (OH) was found (Engvall et al. 1991).

Many individuals with DM1 have a medical condition that require special care. Cardiomyopathy, respiratory failure, dysphagia, and daytime sleepiness are common. They have an increased sensibility for certain anesthetics and need well‐planned perioperative care (Harper 2001; Rogers & Clyburn 2004).

Sjögreen et al. have shown that access to prophylactic treatment and supportive specialized dental care could be important in order to prevent oral disease (Sjögreen et al. 2015), in addition to good dietary habits. Children with intellectual disability or other neurodevelopmental disorders presenting with dental fear can be familiarized with the dental situation through frequent, individually adjusted visits to the dental clinic (Valle‐Jones & Chandler 2015; Hamzah et al. 2014). In addition to professional support, individuals with disability may need daily support with oral care in order to maintain good OH (Mac Giolla Phadraig et al. 2013). Assistive devices for oral care, such as bite support, mouth angle expanders, cushions for sitting support, xerostomia treatment products, and special toothbrushes and toothpastes, can facilitate oral care and may result in improved oral health (da Mata et al. 2009; Gabre et al. 2013; Dogan et al. 2004).

There is however, little knowledge of the risk factors and protective factors for oral health in patients with DM1. In the present study, we have therefore performed an in‐depth examination in order to identify these factors, making it possible to guide clinicians on how to offer the effective prevention of dental diseases in this medically compromised patient group.

The primary aim of the present study was to explore the interaction between OH, oral care, saliva production, and oral motor function in individuals with the congenital and the childhood‐onset forms of DM1. A secondary aim was to study how OH, oral care, and saliva flow rate are affected by gender, age, and subgroup of DM1 in this patient group.

## Methods

### Study population

Eighty individuals (0–29 years) with a congenital or childhood‐onset form of DM1 living in western and southern Sweden were invited by a pediatric neurologist to participate in the third part of a longitudinal, multidisciplinary, epidemiological study, and 71 accepted the invitation. Of these, 52 individuals, aged seven to 29 years (mean age 18.4 years), participated in the present study of OH aspects. They were divided into two subgroups of DM1, the congenital (*N* = 24) and childhood‐onset forms (*N* = 28), and three age groups (Table 1). Nineteen of the individuals who took part in the multidisciplinary study were not included because of young age (<3 years) (*n* = 4) or their inability to co‐operate in the examination because of a severe neurodevelopmental disorder or illness (*n* = 11) and four did not want to participate in the dental examination.

**Table 1:** Description of the study group in a study of 52 children and young adults with DM1. Age group 1 = 7–12 years, age group 2 = 13–19 years, and age group 3 = 20–29 years.

DM1 subgroup	No (m/f)	Median age
**Whole group**	52 (27/25)	18
*Age group 1*	11 (6/5)	11
*Age group 2*	18 (11/7)	17
*Age group 3*	23 (10/13)	23
**Congenital**	24 (13/11)	19
*Age group 1*	5 (3/2)	12
*Age group 2*	7 (5/2)	17
*Age group 3*	12 (5/7)	23
**Childhood onset**	28 (14/14)	17.5
*Age group 1*	6 (3/3)	10.5
*Age group 2*	11 (6/5)	17
*Age group 3*	11 (5/6)	25

### Procedure

This part of the multidisciplinary study of DM1 focuses on OH and the results of the examinations made by a dentist and a speech‐language pathologist in co‐operation. Examinations were made at dental clinics close to where the patients lived.

Informed consent was obtained from each family and the study was approved by the Ethics Committees at the Medical Faculties at Gothenburg and Lund Universities.

#### Dental examination

Indices for the occurrence of calculus, gingivitis, and plaque were created from the examination of six selected teeth according to Ramfjord (1967). An OH grading scale was constructed based on the Ramfjord index scale for calculus, plaque, and/or gingivitis. Individuals with a calculus‐, gingivitis‐, or plaque‐index score of 5–6 were considered to have poor OH, 3–4 fair OH, and 0–2 good OH. The number of decayed, missing, and filled permanent teeth (DMFT) were clinically examined and completed with bitewings. Only manifest caries spreading into the dentine was registered.

Four dental cotton rolls (IVF Hartmann AG, CH‐8212 Neuhausen/Switzerland, 8 mm Ø) connected with dental floss were used to measure the unstimulated whole saliva flow rate (Møller et al. 2015; Rotteveel et al. 2004). Two were placed in the vestibules of the upper jaw and two under the tongue while the patient was seated in a dental chair in an upright position. Saliva was collected for 3 min and the cotton rolls were weighed (KERN PCB, *d* = 0.001 g) before and after use. The procedure was repeated twice and the saliva flow rate per minute was calculated from the mean value. An unstimulated whole saliva flow rate of ≤0.3 mL/min was considered low (Rotteveel et al. 2004; Wolff & Kleinberg 1998).

#### Oral motor assessment

Oral motor function was assessed by a speech‐language pathologist according to a standardized protocol (Holmberg & Bergström 2008) and video‐recorded (Sony Handycam, 3CCD Mega Pixel, Sony Corporation, Tokyo, Japan). This included an assessment of the mobility of the facial and tongue muscles. The resting position of the lips, jaw, and tongue was observed for 1 min. Each variable was scored on a four‐point scale and, for the purpose of the present study, dichotomized to no/mild impairment or moderate/severe impairment.

#### Questionnaire

The parents and/or the patient answered questions about general health and disabilities, medication, dental care, and oral health. They were asked if they needed help with toothbrushing, what kind of dental care they received, how often they visited the dental clinic, and whether they had any difficulties with a dry mouth. Dietary habits were estimated on a question about how often they drank sweetened drinks.

### Reliability

The bitewing radiographs were analyzed by two dentists included in the study. The examiners were blinded to the identity of the patient and the inspections were made separately. In the event of disagreement, a consensus decision was made after a discussion and re‐evaluation.

### Statistical analysis

IBM SPSS statistics 22 was used for non‐parametric correlation analyses (Kendall's tau_b) and for the study of differences (cross‐tabulations) between subgroups of DM1, age groups, and gender. Results from the cross tabulations were presented as Pearson Chi‐Square (χ2) statistics and *p*‐values. A *p*‐value ≤0.5 was considered as significant.

## Results

### OH index scores

Examination revealed that more than four of the six Ramfjord teeth (index score 5–6) were affected by calculus in 1.9%, by gingivitis in 17.3%, and by plaque in 27% of the study group (Table 2). There were no significant differences between age groups in terms of calculus (χ2 = 6.361, *p* = 0.384), gingivitis (χ2 = 10.238, *p* = 0.595), or plaque (χ2 = 13.308, *p* = 0.347). Nor were there any significant gender differences in terms of calculus (χ2 = 2.952, *p* = 0.399), gingivitis (χ2 = 8.328, *p* = 0.215), or plaque (χ2 = 7.474, *p* = 0.279) or between subgroups of DM1 (χ2 = 2.428, *p* = 0.489), gingivitis (χ2 = 9.134, *p* = 0.166), or plaque (χ2 = 1.861, *p* = 0.932). Twenty individuals had good OH, 16 fair, and 16 poor OH.

**Table 2:** The occurrence of gingivitis, plaque, and DMFT (Decayed‐Missed‐Filled‐Teeth) in 52 individuals with DM1. Age group 1 = 7–12 years, age group 2 = 13–19 years, and age group 3 = 20–29 years. The Gingivitis Index and Plaque Index are based on the Ramfjord index scale 0–6 (Ramfjord 1967).

**DM1 subgroup**	**Gingivitis Index** Number (%)			**Plaque Index** Number (%)			**DMFT score** Number (%)				
	**0–1**	**2–4**	**5–6**	**0–1**	**2–4**	**5–6**	**0**	**1–3**	**4–6**	**7–9**	**>9**
**Whole group**	21 (40)	22 (42)	9 (17)	16 (31)	22 (42)	14 (27)	19 (37)	11 (21)	9 (17)	8 (15)	5 (9)
*Age group 1*	7	2	2	5	6	0	10	1	0	0	0
*Age group 2*	4	10	4	4	7	7	5	7	3	2	1
*Age group 3*	10	10	3	7	9	7	4	3	6	6	4
**Congenital**	11 (46)	8 (33)	5 (21)	8 (33)	10 (42)	6 (25)	9 (38)	2 (8)	5 (21)	4 (17)	4 (17)
*Age group 1*	4	1	0	2	3	0	5	0	0	0	0
*Age group 2*	3	2	2	2	4	1	2	1	2	1	1
*Age group 3*	4	5	3	4	3	5	2	1	3	3	3
**Childhood**											
**onset**	10 (36)	14 (50)	4 (14)	8 (29)	12 (43)	8 (29)	10 (36)	9 (32)	4 (14)	4 (14)	1 (4)
*Age group 1*	3	3	0	3	3	0	5	1	0	0	0
*Age group 2*	4	6	1	2	3	6	3	6	1	1	0
*Age group 3*	3	5	3	3	6	2	2	2	3	3	1

### DMFT

DMFT score = 0 in one third (36%) of both the congenital and the childhood‐onset form of DM1. Manifest caries was detected in ten individuals (19%). There was a significant correlation between age and the DMFT score (t_b_ = 0.442, *p* < 0.001). DMFT also correlated significantly with the OH grading scale (t_b_ = 0.413, *p* < 0.001).

### Oral care

Almost all patients reported that they received excellent (75%) or good (17.3%) dental care. Dental‐care visits three or more times a year were common (46.9%) and the rest received dental care once (32.7%) or twice (14.3%) a year. Only three individuals (6.1%) visited the dental clinic less than once a year. Furthermore, they thought that they were able to manage their OH to a high degree (42.3%) or to some degree (51.9%). Four patients (6%) brushed their teeth once a day and the rest twice a day or more and they all used toothpaste regularly. Half the study group always brushed their teeth without help, a quarter sometimes received help, and a quarter always received help with toothbrushing. Individuals who were dependent on help with toothbrushing were found in all age groups. Help with toothbrushing was more common in the subgroup of individuals with the congenital form of DM1 compared with the group with the childhood‐onset form (χ2 = 14.641, *p* = 0.006). Nine of 16 individuals with poor OH (56%) never received help with toothbrushing (Table 3). Eight individuals with DM1 (16%) answered in the questionnaire that they drank sweetened drinks several times a day, 22 (44%) almost every day, and 20 (40%) once a week or less. The occurrence of caries (DMFT > 0) correlated with a higher consumption of sweetened drinks (χ2 = 7.801, *p* = 0.020).

**Table 3:** The relationship between oral hygiene and selected reported or examined parameters expected to increase or reduce the risk of having calculus, plaque, and gingivitis.

	**OH grading scale**				
	**Poor** a (*n* = 16) No (%)	**Fair** b (*n* = 16) No (%)	**Good** c (*n* = 20) No (%)	**χ2**	***P***
**Results of examination:**					
Unstimulated whole saliva flow rate ≤ 0.3 mL/min	3 (18.8)	1 (7.7)	2 (11.8)	0.812	0.666
Moderately–severely impaired mobility of the facial muscles	10 (62.5)	10 (62.5)	13 (65.0)	0.033	0.984
Moderately–severely impaired mobility of the tongue	2 (12.5)	3 (18.8)	4 (20.0)	0.383	0.826
**Results of questionnaire:**					
Special care dentistry	7 (43.8)	5 (33.3)	3 (15.8)	3.347	0.188
To a high degree satisfied with dental care	8 (57.1)	15 (93.5)	16 (84.2)	6.568	0.037 *
To a high degree satisfied with oral care at home	4 (28.6)	5 (31.3)	13 (65.0)	5.988	0.050 *
Toothbrushing without help	9 (60.0)	10 (62.5)	8 (42.1)	1.765	0.414

a Poor OH > 4,

b Fair OH = 3–4 and

c Good OH = 0–2, based on the Ramfjord index scale for calculus, plaque, and/or gingivitis.

### Oral clearance

**Figure 1:**
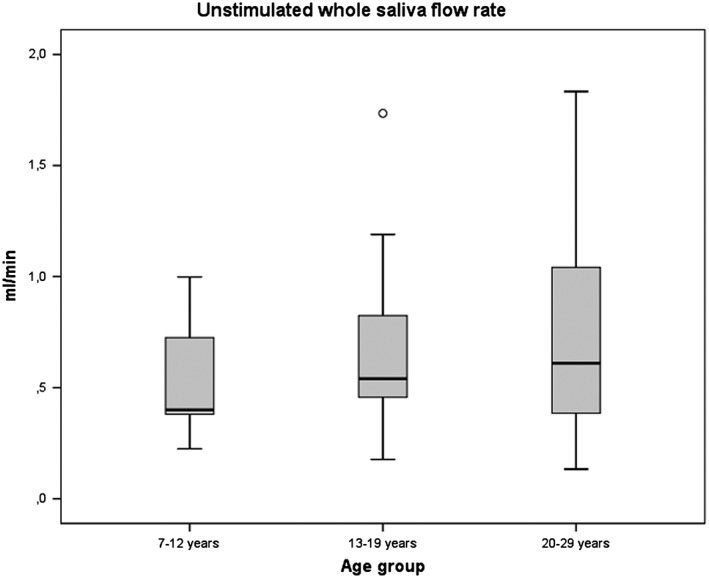
Unstimulated whole saliva flow rate (mL/min) in three age groups of individuals with DM1.

The mean value of the unstimulated whole saliva flow rate was 0.7 mL/min (±0.44) in the study group. In the youngest age group, the saliva flow rate was lower and the spread was more limited in children and teenagers compared with adults (Fig. 1). However, there was no significant correlation between the unstimulated whole saliva flow rate per minute and age (t_b_ = 0.037, *p* = 0.725). Dryness of the mouth was reported by seven patients (13.7%), five teenagers, and two adults. Their measured results were 0.26, 0.32, 0.35, 0.4, 0.77, and 1.19 mL/min (one missing). No significant correlation was found between the unstimulated whole saliva flow rate and the DMFT score (t_b_ = −0.006, *p* = 0.954).

An open mouth at rest, with an open jaw and low tongue position, was a frequent finding (63.5%). The majority (63.5%) had moderately to severely impaired mobility of the facial muscles (including the lips). Moderately to severely impaired tongue mobility was less common (17.3%).

### Other factors that might influence OH

The relationship between the OH scale and selected reported or examined parameters expected to increase or reduce the risk of having calculus, plaque, and gingivitis was analyzed (Table 3). A significant correlation was found between OH and a high degree of satisfaction with dental care received and also with a high degree of satisfaction with oral care at home.

## Discussion

The increased occurrence of plaque and gingivitis in individuals with DM1 that has been presented in earlier studies (Engvall et al. 2007; Engvall et al. 2009; Engvall et al. 1991; Engvall & Birkhed 1997) was confirmed in the present study. Calculus was seldom found. The occurrence of good, fair, and poor OH was evenly spread and could not be related to age groups, gender, or sub‐groups of DM1.

The relatively high frequency of manifest or restored caries in the study group could possibly be explained by difficulties with personal OH procedures because of diagnosis‐specific hand‐grip impairment (Phillips & Mathieu 2004), lack of self‐insight and motivation, dietary habits, and inefficient oral clearance. In the event of excessive daytime sleepiness and somnolence, a symptom common in DM1 (Laberge et al. 2013), individuals might need frequent meals, rich in carbohydrates, in order to obtain enough energy to stay awake. In the present study, the increased risk of caries development in the study group could not be related to dry mouth.

More than half those with poor OH brushed their teeth by themselves. Even though they brushed their teeth twice a day and used toothpaste, they did not succeed in getting a clean mouth. This indicates that the way they brush their teeth is not efficient enough. In order to improve OH, this group of patients should either receive help with toothbrushing *or* learn a more efficient technique for cleaning their teeth – or both. The results of the present study did not reveal whether they brushed their teeth by themselves because they did not want help or if they were not able to receive help. The results also indicated that obtaining help with toothbrushing is not a guarantee of good OH. This shows that the person who assists with toothbrushing could also make use of instructions and support from dental professionals.

This study was unable to confirm that factors expected to affect oral clearance, such as oral motor dysfunction and a low saliva flow rate, are significantly related to OH and oral health. The results suggest that difficulties with oral clearance could be compensated for by excellent oral and dental care.

### Study limitations

Only individuals who were able to co‐operate in the dental and oral motor examination were included in the present study. The decision relating to participation was made after discussions with the caregivers. The selection of patients might have influenced the results.

The normal procedure for measuring the unstimulated whole saliva flow rate is not adjusted for individuals with muscle weakness and neurodevelopmental disorders and an alternative method, not yet fully validated, therefore had to be used (Navazesh & Kumar 2008). The cotton rolls used for collecting saliva might have had a stimulating effect on the saliva production.

### Clinical implications

Individuals with DM1 who are severely affected by the disease can still have good OH. Regular support from the dental team is fundamental to maintaining good oral health in this group of patients. Individuals with DM1 who have poor OH should be identified and receive extra prophylactic dental care. DM1 is a progressive disease with a high risk of developing cardiac disease, respiratory dysfunction, and dysphagia and the importance of a healthy mouth is therefore of extra importance (Sjögren et al. 2008). In addition, there are certain risks related to general anesthesia (Rogers & Clyburn 2004), which makes preventive oral care highly justified in patients who will require some kind of sedation in connection with dental treatment.

## Conclusions

Many children and young adults with the congenital or childhood form of DM1 have poor OH, with a high occurrence of plaque and gingivitis. As a group, individuals with DM1 and poor OH have a higher frequency of DMFT and they report less satisfaction with their oral care at home and the quality of dental care received compared with those with good OH.

## Declaration of Interest

The authors report no conflicts of interest. The authors alone are responsible for the content and writing of the paper.
